# Detecting Facial Region and Landmarks at Once via Deep Network †

**DOI:** 10.3390/s21165360

**Published:** 2021-08-09

**Authors:** Taehyung Kim, Jiwon Mok, Euichul Lee

**Affiliations:** 1 Department of AI & Informatics, Graduate School, Sangmyung University, Seoul 03016, Korea; 201831036@sangmyung.kr (T.K.); 202032013@sangmyung.kr (J.M.); 2 Department of Human-Centered Artificial Intelligence, Sangmyung University, Seoul 03016, Korea

**Keywords:** single-shot detection, face detection, face landmark detection, simultaneous detection, deep network

## Abstract

For accurate and fast detection of facial landmarks, we propose a new facial landmark detection method. Previous facial landmark detection models generally perform a face detection step before landmark detection. This greatly affects landmark detection performance depending on which face detection model is used. Therefore, we propose a model that can simultaneously detect a face region and a landmark without performing the face detection step before landmark detection. The proposed single-shot detection model is based on the framework of YOLOv3, a one-stage object detection method, and the loss function and structure are altered to learn faces and landmarks at the same time. In addition, EfficientNet-B0 was utilized as the backbone network to increase processing speed and accuracy. The learned database used 300W-LP with 64 facial landmarks. The average normalized error of the proposed model was 2.32 pixels. The processing time per frame was about 15 milliseconds, and the average precision of face detection was about 99%. As a result of the evaluation, it was confirmed that the single-shot detection model has better performance and speed than the previous methods. In addition, as a result of using the COFW database, which has 29 landmarks instead of 64 to verify the proposed method, the average normalization error was 2.56 pixels, which was also confirmed to show promising performance.

## 1. Introduction

The technique of facial landmark detection includes locating a face in an image and obtaining a key point of the face, and many studies employing faces [1], such as facial recognition, face verification, and face 3D modeling, all rely on this process [2,3,4]. Wearable devices are becoming increasingly popular, and there are many ways to interact with other smart devices. A wearable device requires an IoT-based sensing technology that can measure the physiological and behavioral characteristics of users. The incorporation of biometric authentication, through face and iris, into wearable devices is being investigated [5,6]. In addition, goggle-shaped wearable devices for augmented reality (AR) experiences are equipped with a camera that shoots forward [7]. Images taken by the mounted camera can be used for various applications, such as recognizing facial expressions and face correction through face recognition of others [8,9]. Even kiosks equipped with cameras, not wearable devices, can capture the faces of various users and provide interactive and intelligent services through a natural user interface (NUI), such as in healthcare applications and eye tracking [10,11]. Thus, technologies that use face information in the fields of wearable devices and IoT applications are diverse. This necessitates the accurate detection of the facial region and automatic selection of feature points. Face detection with high accuracy is challenging in the actual world due to obstacles such as variable pose, illumination, and occlusion.

Holistic methods, constrained local model (CLM)-based methods, and regression-based methods are conventional facial landmark detection algorithms that use facial appearance and shape information. These three methods differ in terms of seeking and utilizing information. Subsequently, each method will be described as follows.

To recognize face landmarks, holistic methods explicitly utilize the global facial appearance as well as global facial shape patterns. The classic model of holistic methods, the active appearance model (AAM), constructs an overall facial shape model and a global facial appearance model based on the principal component analysis (PCA). The trained appearance and shape models are fitted to the test image to identify the location of the facial landmarks. Therefore, holistic methods focus on improving fitting algorithms. However, fitting algorithms have a trade-off between speed and accuracy. The AAM was found to exhibit a low performance in cross-database experiments [12]. For this reason, lately, AAM-based methods are rarely used, and it is difficult to present a performance comparable to that of other methods that have been recently reported.

CLM-based methods build a model explicitly using the local facial appearance and global facial shape patterns. This refers to the location of a landmark based on the independent local appearance and global shape around each landmark. Face detection, pose estimation, and landmark localization (FPLL) is a unified model for face detection, pose estimation, and landmark estimation [13]. Discriminative response map flitting (DRMF) is a new discriminative regression-based method of CLM, which is very efficient and can be calculated in real time [14]. Tzimiropoulos and Pantic optimized the model through the Gauss-Newton method to solve the limitation of detection ambiguity of deformable part models (DPM). That is, it achieved computational reduction by optimizing the cost function [15]. In case of CLM-based methods, they have an accuracy–robustness trade-off, wherein the accuracy of landmark localization is diminished when utilizing a large local appearance [16].

Next, the regression-based face landmark detection method can be categorized into three types, such as direct, cascaded, and deep-learning-based regression methods. Regression-based methods use local or global facial appearances. Unlike holistic and CLM-based methods, they use an implicit facial shape and directly learn the mapping of the landmark position in the image. Since the direct regression methods learn to map the location of facial landmarks directly from an image, a one-step prediction is performed. Conversely, cascaded regression methods update the landmark location step-by-step using various regression functions to predict the location of the landmark on the face. Recently, the detection of deep-learning-based face landmarks has been carried out using the convolutional neural network (CNN) model. Regression-based face landmark detection methods have been published in several studies. A method called robust cascaded pose regression (RCPR) to detect occlusion and reduce external exposure achieved 80% precision by presenting a new database called Caltech occluded faces in the wild (COFW) [17]. The supervised descent method (SDM) is a method to minimize the non-linear least squares (NLS) function and shows good performance in the facial feature detection problem [18]. Coarse-to-fine auto-encoder networks (CFAN) can predict landmarks accurately and quickly by cascading stacked auto-encoder networks (SAN) [19]. Coarse-to-fine shape searching (CFSS) is a new face alignment framework that prevents being trapped in the local optimum by starting a coarse search for a space containing various face shapes [20]. It is robust to large pose variations. However, such regression-based methods have the disadvantage that trained algorithms may not work appropriately. In other words, a face region detection model that is different from the trained algorithm may not correctly predict the landmarks [21]. Since these are methods of detecting landmarks after face region detection, they are inevitably dependent on the face region detection result. Statistical performance and accuracy will be compared with our proposed method in the experimental results.

Therefore, depending on the facial region detection model, the landmark detection performance is affected. Landmark extraction is a basic function in tasks such as facial recognition and facial expression detection; therefore, it must be performed consistently in a variety of settings. This requires a face landmark detection model that is unaffected by the face region detection model.

In this paper, we propose a single-shot face landmark detection model to solve the shortcomings of prior methods. The proposed method has the following advantages: (1) the proposed model is unaffected by the performance of the face region detection model, (2) it can accurately and quickly detect faces, and (3) it performs well in terms of generality.

## 2. Materials and Methods

**Figure 1:**
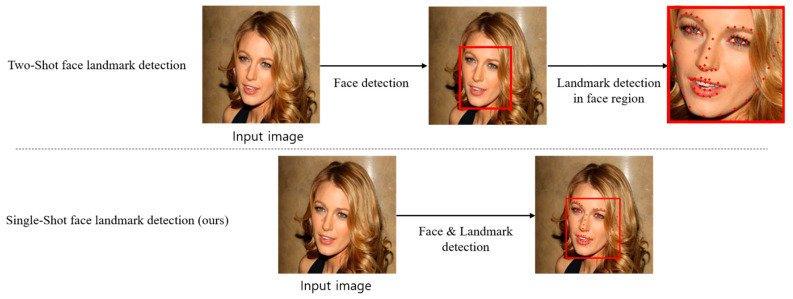
Comparison between a prior method and the proposed method for detection of facial landmarks.

As shown in Figure 1, in the previous face landmark detection model, an input image is inputted to a face region detection model, which returns bounding box coordinates for the face region. The bounding box coordinates are then used to crop the face region, which is subsequently inputted to a face landmark detection model to obtain the landmark coordinates. Clearly, there are two steps in this method: face region detection and landmark detection. As mentioned previously, landmark detection is influenced by the face region detection model. In addition, the prediction speed is inconsistent because the number of face landmarks that must be detected is as many as the number of faces in the image.

To effectively address these issues, we propose a single-shot face landmark detection model. Unlike previous methods, which require two steps, the method we have proposed can detect facial regions and landmarks at the same time with only one model. Therefore, good performance and high speed can be ensured by selecting only the backbone network for feature extraction.

### 2.1. Architecture of the Proposed Model

The method proposed in this paper was inspired by the method reported in [22]. In [22], a 40 × 40 gray image is input to the proposed network, which then goes through a feature extraction step with four convolution layers. After that, a multi-tasks feature vector is created and multiple regression values are calculated. Given that the image of the face region is inputted, detection of the face region must be prioritized. This can also affect the performance of landmark detection because it detects landmarks after facial region detection. Therefore, we propose a single-shot detection model that simultaneously performs landmark detection and face region detection based on the 1-stage object detection model.

**Figure 2:**
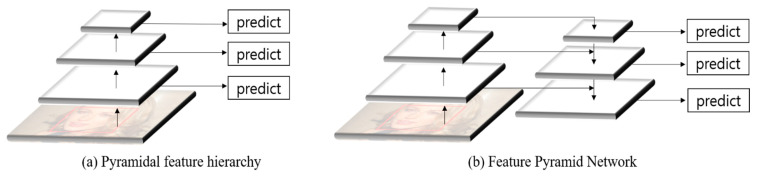
Structure of the feature map used in SSD and YOLOv3: (**a**) Pyramidal feature hierarchy method used in SSD, and (**b**) feature pyramid network method similar to that used in YOLOv3.

You Only Look Once v3 (YOLOv3) [23] and Single-Shot MultiBox Detector (SSD) [24] are widely used as 1-stage object detection models. Although both models have similar accuracy, the YOLOv3 is faster. SSD utilizes pyramidal feature hierarchy, an object detection method that creates feature maps of multiple scales from the input image and extracts features independently at each scale. This is suitable for detecting an object with appropriate semantics in each map represented by various resolutions, as shown in Figure 2a. However, since a predictor suitable for the specific resolution of each map operates independently from the predictor of other maps, it is difficult to adaptively operate at various resolutions. YOLOv3 utilizes an approach comparable to that of the feature pyramid network (FPN) [25]. A method of combining high-level and low-level features using the skip connection concept is incorporated into the pyramidal feature hierarchy method. Therefore, it can have strong semantics at all scales and is robust to various scale changes. Figure 2 shows the methods used in SSD and YOLOv3. YOLOv3, which can use powerful semantics, was chosen as the base model because it can more effectively find faces of various scales.

**Figure 3:**
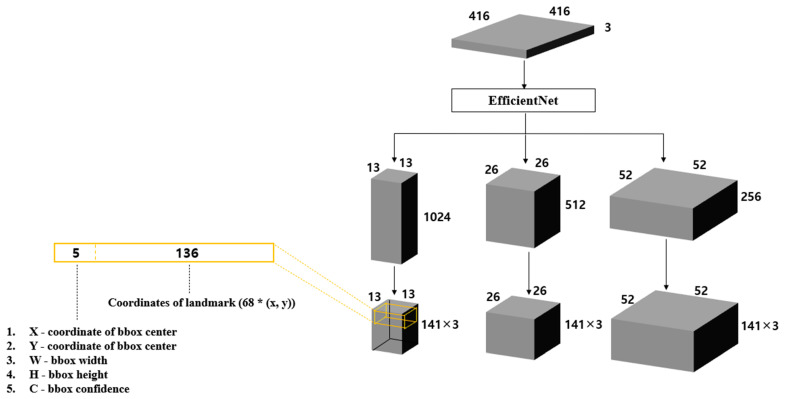
Structure of the proposed model.

The proposed model differs from the original YOLOv3 model in that it predicts the coordinates of the landmark rather than predicting the class of the object in the bounding box. In the original YOLOv3, when the input image is divided into S × S cells, each cell contains information about the bounding box and class probability. We modified the model to leave the information about the bounding box and added the coordinates of the landmark instead of the class probability. If an input image of size 416 × 416 × 3 is inputted to the model, feature maps with three scales, 52 × 52 × 256, 26 × 26 × 512, and 13 × 13 × 1024, are outputted. When the number of landmarks is 68, each cell has 141 parameters by adding 136 coordinates of the landmark and five pieces of information about the bounding box. Therefore, to extract the face region and face landmark information, each feature map is matched to a 141 × 3 channel. Figure 3 shows the structure of the proposed model.

### 2.2. Loss Function of the Proposed Model

The loss function for training was also based on the one used in YOLOv3. The original method uses a combination of localization loss, confidence loss, and classification loss. The classification loss is calculated using the squared error. If there is an object in the corresponding cell, the term $1_{ij}^{obj}$ becomes 1, and if there is no object, it becomes 0. The classification loss equation is as follows:

(1) $$\overset{S^{2}}{\underset{i=0}{\sum }}1_{ij}^{obj}\underset{c\in classes}{\sum }{\left(p_{i}\left(c\right)-{\hat{p}}_{i}\left(c\right)\right)}^{2}$$

For single-shot face landmark detection, we remove Equation (1), which is the classification loss, and add the sum squared error (SSE), as expressed in Equation (2). We call Equation (2) the landmark loss. Likewise, if no object is detected in the corresponding cell, $1_{ij}^{obj}$ will be 0. $\lambda_{coord}$ is a weight added to improve the detection performance. The size of the feature map is $S^{2}$ (width × height), and *B* is the number of anchor boxes. *X* and *Y* represent the coordinates of the landmarks.

(2) $$\lambda_{coord}\overset{S^{2}}{\underset{i=0}{\sum }}\overset{B}{\underset{j=0}{\sum }}1_{ij}^{obj}\left[{\left(x_{i}-{\hat{x}}_{i}\right)}^{2}+{\left(y_{i}-{\hat{y}}_{i}\right)}^{2}\right]$$

As a result, the entire loss of the proposed model is a combination of the localization, confidence, and landmark losses.

### 2.3. Backbone Network

The backbone network has a considerable impact on the performance and processing speed of the CNN-based model. In the original YOLOv3, Darknet-53 was used as the backbone network. This network was built as a deep network to increase the accuracy of multiclass classification. In this study, only one type of human face was used. Therefore, to increase the processing speed, various backbone networks were compared and analyzed, and the best backbone network was selected. The backbone networks compared were EfficientNet-B0 [26], MobileNetv1 [27], and ResNet50 [28]. All three backbone networks were trained in the same GPU environment. We selected EfficientNet-B0 as the backbone network as it showed the best performance. A detailed performance comparison between the backbone networks is described in Section 3.2.

**Figure 4:**
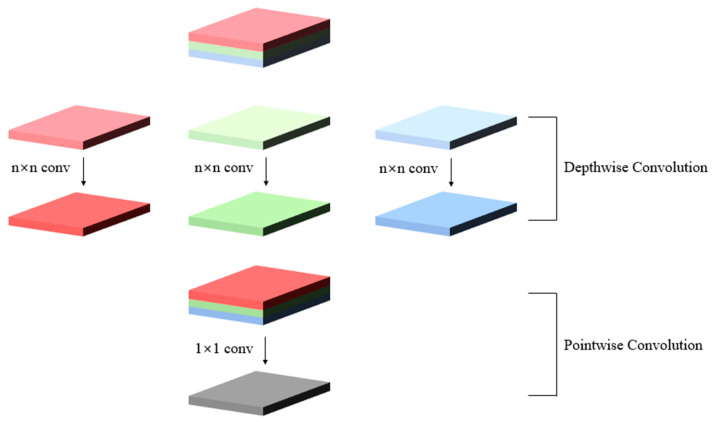
Structure of depth-wise separable convolution of EfficientNet.

In EfficientNet, a depth-wise separable convolution procedure, which combines depth-wise convolution and pointwise convolution, is applied. Convolution operation utilizing each kernel by splitting each channel is known as depth-wise convolution, and the input and output channels are always the same. Pointwise convolution is a convolution operation that uses a 1 × 1 kernel to modify the output channel. This can drastically reduce the number of parameters, which increases the speed, and the performance does not decrease significantly. Figure 4 shows the depth-wise separable convolution method. In addition, a compound scaling method is used to improve the model performance. Previously, methods for improving the performance of the convolution network involved simply deepening the network or widening it, or changing the size of the model based on the image resolution. EfficientNet shows good performance through the compound scaling method, which scales the depth, width, and resolution of the network evenly. Therefore, EfficientNet-B0, which is a baseline network in EfficientNet, was used for the single-shot detection model.

### 2.4. Database for Model Training and Verification

**Figure 5:**
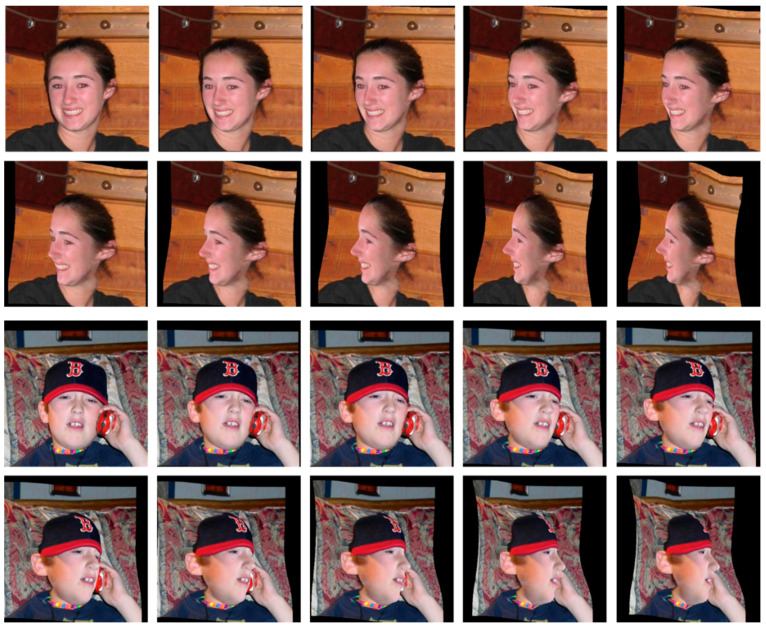
Example image of 300W-LP database.

We used 300 W across large poses (300W-LP) [29] to train the model. The 300W-LP is a database that applies the rotation of 3D images to 300 W [13] for data augmentation, and is a standardized database of 68 landmarks for HELEN [30], LFPW [16], and AFW [13]. The total amount of data for 300W-LP is 122,450. The 300W-LP is composed of images taken from various angles, from the front to the sides, by applying rotation to the human face. Images of one person are included only in the training set or test set. That is, the person used in the training set is not reused in the test set. Figure 5 shows an example image. For training, the data were separated into a training set (97,967) and a validation set (22,035). The evaluation included the remaining data (Helen: 1616, LFPW test set: 832) that were not used for the training.

The COFW [17] database was used as a database to verify the results obtained in this study. The COFW is composed of images with various shapes and occlusions from the real world. The average occlusion of COFW is more than 23%, and has 29 landmarks. The total number of training data for COFW is 1345, and the data are divided into a training set (1076) and a validation set (269). The number of test data for COFW is 507.

### 2.5. Evaluation Metric

The average normalized error was used as an evaluation metric. Since the face region in an image varies based on the object, position, and surrounding, the average normalized error is commonly applied as an evaluation metric [31]. Equation (3) was used to calculate the average normalized error. In Equation (3), *N* is the number of landmarks, and $d_{i}$ is the landmark coordinates predicted by $\left\{d_{x,i},\;d_{y,i}\right\}$. $g_{i}$ is $\left\{g_{x,i},g_{y,i}\right\}$, which correspond to the ground-truth landmark coordinates. $g_{le}$ and $g_{re}$ are normalization indicators that prevent the errors from altering due to transformations in the image or face size. In this study, the inner corners of the left and right eyes were used.

(3) $$\frac{1}{N}\overset{N}{\underset{i}{\sum }}\frac{{\|d_{i}-g_{i}∥}_{2}}{{\|g_{le}-g_{re}∥}_{2}}$$

## 3. Results

### 3.1. Experimental Setup

The database used for training and testing was 300W-LP, and each image has the coordinates of 68 landmarks from one face. The database used for verification is COFW, and each image has the coordinates of 29 landmarks from one face. The size of the input image of the proposed model in this study was 416 × 416. The larger the image size, the higher the detection performance and the lower the inference speed. Therefore, 512 × 512 is generally used when performance is important, and 300 × 300 or smaller is generally used when speed is important. We used a medium size of 416 × 416 because we value both detection performance and inference speed. The learning rate of the model was set to 0.001, and Adam was used as the optimizer. ReduceLROnPlateau was used as the scheduler of the learning rate. Training and testing were performed on a desktop with an NVIDIA GeForce GTX1080Ti (Santa Clara, CA, USA) and 64-bit Windows 10 environment, and these processes were implemented using Python and PyTorch.

### 3.2. Comparison Results of Backbone Network

The performance was compared using three backbone networks, namely EfficientNet-B0, MobileNetv1, and ResNet50, which were trained in the same environment. Table 1 lists the training results of the three backbone networks. The evaluation results of the three backbone networks showed that the normalized errors of EfficientNet-B0, MobileNetv1, and ResNet50 were 2.32, 3.54, and 3.69, respectively. EfficientNet-B0 exhibited the fastest and best performance. Therefore, in this study, EfficientNet-B0 was chosen as the backbone network of the proposed model.

### 3.3. Training Results

**Figure 6:**
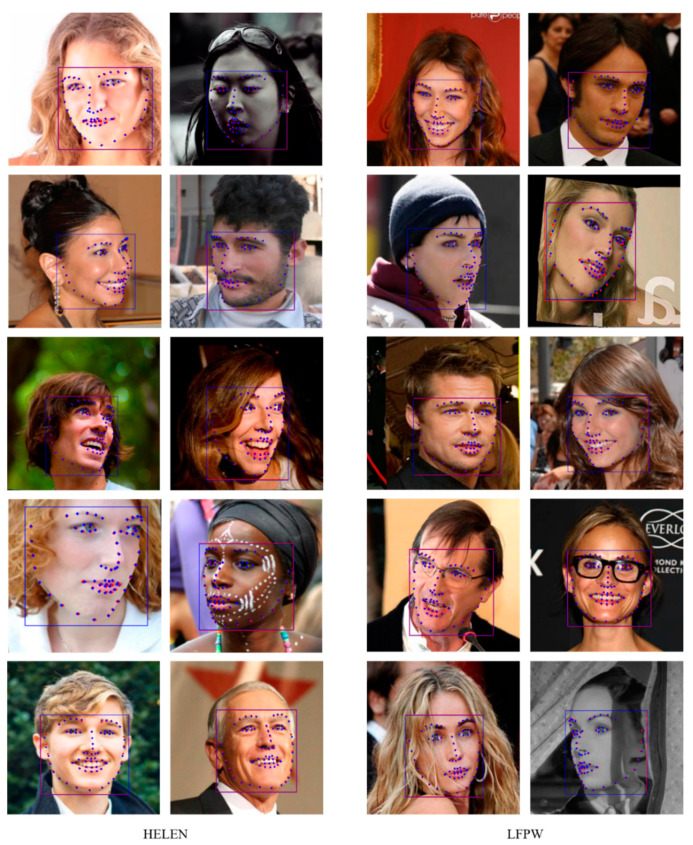
Test results of the proposed model of 68 landmarks. Blue dot: ground truth of face landmarks, Red dot: predicted face landmarks, Blue box: ground truth of face region, Red box: predicted face region.

The training was terminated early at a training loss of 0.0017 and a validation loss of 0.1041. Figure 6 shows the landmark extraction results of the trained model. The processing speed per image was 15 ms. We compared the accuracy of the proposed model quantitatively to prior research that measured the accuracy using HELEN [30] and LFPW [16]. The above-mentioned average normalized error was adopted as the evaluation metric. Since the goal is to calculate the distance difference between the ground truth and the predicted landmark, the general error rate was calculated [31]. The performance of prior methods was reported in [31]. Table 2 lists the evaluation results. The evaluation results for HELEN and LFPW were 2.36 and 2.28, respectively. The lowest normalized error rate was confirmed on both databases. The proposed model allows to extract objects at various scales using FPN, so we were able to see better performance than previous methods.

**Figure 7:**
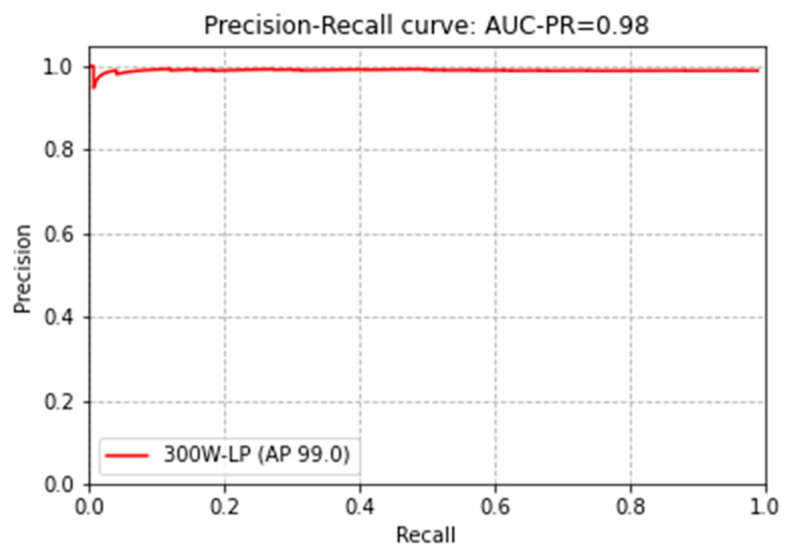
Precision-recall curve of the proposed model on the 300W-LP dataset.

Furthermore, we evaluated the face detection accuracy of the proposed model. The average intersection over union (IOU) of the ground truth face region and the predicted face region for the test set with 2448 images was 0.98, which is quite high in accuracy. Figure 7 shows the precision-recall curve for the 300W-LP dataset of our proposed model. The average precision of 300W-LP achieved 99%.

In addition, to quantitatively confirm the speed of the proposed model, we compared the processing speeds reported in previous studies. Among the previous studies compared in Table 2, we compared it with studies using the regression-based method and 68 landmarks, similar to ours. The regression-based method also includes a deep learning method. The processing speed per image in this study was 67 fps. The processing speed results were compared only with those of previous studies that experimented with 68 landmark points, as listed in Table 3. The processing speed of the previous methods was reported in [31], and the processing speed of our method was higher than those of the other methods. We were able to apply the depth-wise separable convolution of the backbone network to significantly reduce the amount of computation and obtain a fast operation speed.

### 3.4. Verification Results

**Figure 8:**
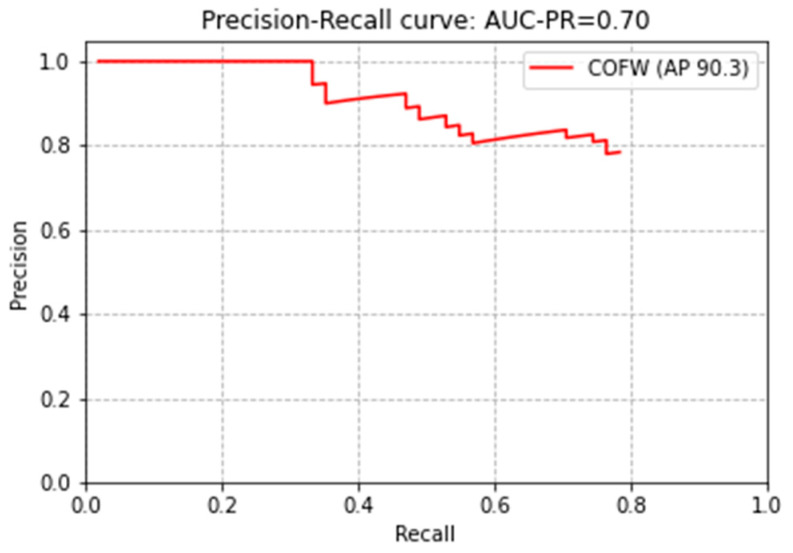
Precision-recall curve of the proposed model on the COFW dataset.

**Figure 9:**
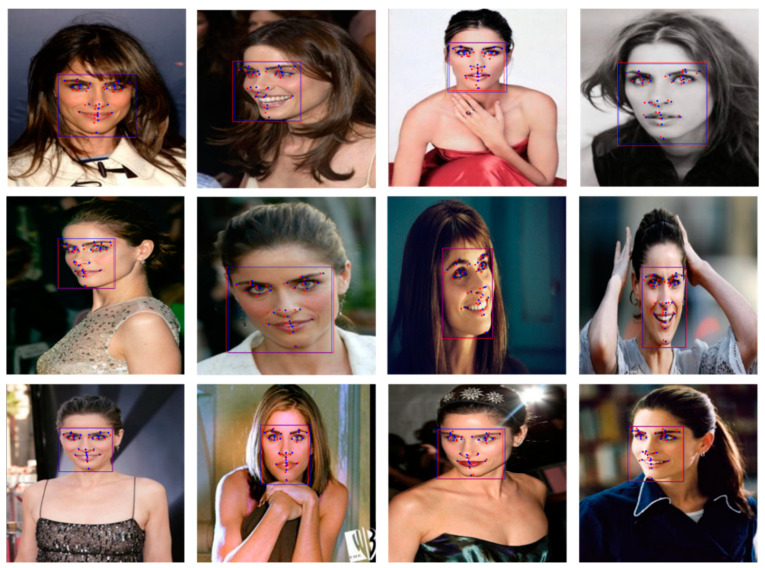
Test results of the proposed model of 29 landmarks. Blue dot: ground truth of face landmarks, Red dot: predicted face landmarks, Blue box: ground truth of face region, Red box: predicted face region.

To verify the results obtained in this study, the COFW database with 29 landmarks was used for model training, not the 300W-LP database with 68 landmarks. The training was conducted in the same environment as the previous training, and EfficientNet was used as the backbone network. The training was stopped early at a training loss of 0.0025 and a validation loss of 0.0963. The result of evaluating the normalized error was 2.56. The processing speed per image was 71 fps. It can be seen that the detection speed of 29 landmarks was slightly faster than the detection speed of 68 landmarks. The average IOU for the test set with 507 images was 0.78, which had high face region detection accuracy. The precision-recall curve for the COFW dataset can be seen in Figure 8. The average precision of COFW was 90.3%. Figure 9 shows the 29 landmark extraction results of the trained model.

## 4. Conclusions

In this study, we established a single-shot face landmark detection model that simultaneously performs face region detection and face landmark detection. There is no need for an additional face detection step. As a result, a consistent detection performance can be expected without being affected by the face detection stage. By comparing three backbone networks, namely EfficientNet-B0, MobileNetv1, and ResNet50 based on YOLOv3, we were able to obtain the best performance and high processing speed owing to the depth-wise separable convolution operation of EfficientNet. In addition, through the compound scaling method of EfficientNet, good performance was obtained by balancing the width, depth, and resolution of the network. To accelerate single-shot detection through a small number of parameters, this study used EfficientNet-B0, the baseline network of EfficientNet designed through a neural architecture search (NAS). Based on YOLOv3 but using EfficientNet as the backbone network, face region detection and face landmark detection could be performed quickly and accurately in one step. Therefore, additional processing, such as gaze tracking, face recognition, and facial expression recognition, is easier to implement.

In the future, we plan to develop a model that can be used in both CPU and mobile environments. For this purpose, we plan to use sophisticated networks such as MobileNetv3. In addition, it is expected that the single-shot face landmark detection model can be used for eye-tracking research and wearable biometric recognition systems.

## Figures and Tables

**Table 1:** Performance results for the three backbone networks studied.

	EfficientNet-B0	MobileNetv1	ResNet50
Training loss	0.0017	0.0039	0.0030
Validation loss	0.1041	0.0847	0.2054
Normalized error	2.32	3.54	3.69

**Table 2:** Comparison of performance with prior methods using average normalized error as the evaluation metric.

HELEN		LFPW	
Method	Normalized Error	Method	Normalized Error
FPLL [13]	8.16	FPLL [13]	8.29
DRMF [14]	6.70	DRMF [14]	6.57
RCPR [17]	5.93	RCPR [17]	6.56
Gaussian–Newton DPM [15]	5.69	Gaussian–Newton DPM [15]	5.92
SDM [18]	5.53	SDM [18]	5.67
CFAN [19]	5.50	CFAN [19]	5.44
CFSS [20]	4.63	CFSS [20]	4.87
Proposed method	2.36	Proposed method	2.28

**Table 3:** Processing speed comparison with previous methods.

Method	fps
SDM [18]	30
CFAN [19]	40
CFSS [20]	25
Proposed method	67

## Data Availability

Only publicly available datasets were used in this study. The data can be found here: (300W-LP) http://www.cbsr.ia.ac.cn/users/xiangyuzhu/projects/3DDFA/main.htm; (COFW) http://www.vision.caltech.edu/xpburgos/ICCV13/ (accessed on 6 August 2021).
